# Looking at the other side of the coin: the search for possible biopositive cognitive effects of the exposure to 900 MHz GSM mobile phone radiofrequency radiation

**DOI:** 10.1186/2052-336X-12-75

**Published:** 2014-04-26

**Authors:** Seyed Ali Reza Mortazavi, Ali Tavakkoli-Golpayegani, Masoud Haghani, Seyed Mohammad Javad Mortazavi

**Affiliations:** 1 Ionizing and Non-ionizing Radiation Protection Research Center (INIRPRC), School of Medicine, Shiraz University of Medical Sciences, Shiraz, Iran; 2 Standard Research Institute, Tehran, Iran; 3 Department of Medical Physics and Medical Engineering, School of Medicine, Shiraz University of Medical Sciences, Shiraz, Iran; 4 Medical Physics & Medical Engineering Department, School of Medicine, Imam Hossein, Shiraz, Iran

**Keywords:** Mobile phone, GSM, Radiofrequency, Non-ionizing radiation, Microwave, Beneficial effects

## Abstract

Although exposure to electromagnetic radiation in radiofrequency range has caused a great deal of concern globally, radiofrequency radiation has many critical applications in both telecommunication and non-communication fields. The induction of adaptive response phenomena by exposure to radiofrequency radiation as either increased resistance to a subsequent dose of ionizing radiation or resistance to a bacterial infection has been reported recently. Interestingly, the potential beneficial effects of mobile phone radiofrequency radiation are not only limited to the induction of adaptive phenomena. It has previously been indicated that the visual reaction time of university students significantly decreased after a 10 min exposure to radiofrequency radiation emitted by a mobile phone. Furthermore, it has been revealed that occupational exposures to radar radiations decreased the reaction time in radar workers. Based on these findings, it can be hypothesized that in special circumstances, these exposures might lead to a better response of humans to different hazards. Other investigators have also provided evidence that confirms the induction of RF-induced cognitive benefits. Furthermore, some recent reports have indicated that RF radiation may play a role in protecting against cognitive impairment in Alzheimer’s disease. In this light, a challenging issue will arise if there are other RF-induced stimulating effects. It is also challenging to explore the potential applications of these effects. Further research may shed light on dark areas of the health effects of short and long-term human exposure to radiofrequency radiation.

## Introduction

Non-ionizing Radiation is a part of the electromagnetic radiation (EMR) which due to its lower energy is unable to produce ionization. However, non-ionizing radiation affects the cells electrically, chemically and thermally causing a wide range of beneficial or harmful effects. The radiofrequency electromagnetic radiation (RF EMR) component of the electromagnetic radiation which is produced by both natural and artificial sources can be defined as that part of the spectrum where electromagnetic waves have frequencies in the range of about 3 kHz to 300 GHz [1].

The strength of the RF field is usually expressed in terms of its two cardinal components, electric and magnetic fields. The strength of electric and magnetic field components of RF EMR are measured in units of V/m and A/m, respectively. On the other hand, the “power density” can also be used to characterize an RF field. Power density that is expressed in units of W/m^2^ can be defined as power per unit area. On the other hand, specific absorption rate (SAR), that is usually expressed in units of W/kg, is often used to measure the amount of RF radiation absorbed in the body [2]. Specific absorption rate is usually averaged either over the whole body or over a small sample volume (typically 1 g or 10 g of tissue). When the electric field is known, SAR can be calculated within the tissue as:

$$\mathrm{SAR}=\int_{\mathrm{sample}}\frac{\sigma \left(r\right)|E\left(r\right)|{}^{2}}{\rho \left(r\right)}dr$$

Where σ is the sample electrical conductivity, E is the root mean square (RMS) electric field, and ρ is the sample density. According to WHO, the minimum SAR that is needed to produce known adverse health effects in humans exposed to RF in the frequency range of 1 MHz to 10 GHz is about 4 W/kg [3]. RF EMR has many applications in both telecommunication (mobile phones, cordless phones, wireless computer networks, radio and television broadcasting, satellite communications) and non-communication (microwave ovens, industrial RF heating and sealing) fields. Despite these applications, there are reports indicating higher risk of tumor formation in heavy mobile phone users [4-7]. It has also been claimed that health symptoms such as tiredness, stress, headache, anxiety, concentration difficulty and sleep disturbance are frequently reported by the users of mobile phones [8-10]. However, Mortazavi et al. found no significantly higher prevalence of self-reported symptoms in individuals who had used mobile phones [11]. On the other hand, some recent studies were unable to show an association between cancer and mobile phone use [12] or living nearby mobile base stations [13]. There was also no association between risk of early childhood cancers and mother’s exposure to mobile phone base stations during pregnancy [14]. A recent study even indicated that short-term exposure to weak microwave radiation can temporarily stimulate specific humoral or cellular immune responses, while prolonged exposures may inhibit the same functions [15]. In this light, it can be concluded that current findings are complicated by a wide range of confounding factors and hence these studies do not show strong and convincing evidence that there is a causal association between cancer and exposure to RF energy [16].

Over the past years, our laboratory has focused on studying the health effects of exposure of laboratory animals and humans to some common and/or occupational sources of electromagnetic fields such as mobile phones [17-24] and their base stations [25], mobile phone jammers [26], laptop computers [27], radars [18], dentistry cavitrons [28] and MRI [29]. To the best of our knowledge this paper is the first article that reviews the beneficial effects of exposure to mobile phone radiofrequency radiation.

### Are there known detrimental effects associated with exposure to RF EMR?

There is growing serious concern that the exponentially increased exposure to RF-EMF from mobile phones might lead to adverse health effects [30]. Cell phones are popular communication devices that emit low levels of RF-EMF. Even in stand-by mode, mobile phones emit a very short signal at certain intervals. Over the past two decades, hundreds of worldwide studies have been conducted to assess the biological effects of RF-EMF. It has been reported that self-reported symptoms such as headache, earache, and warmth sensation, concentration problem and fatigue are associated with using mobile phones [31,32]. On the other hand, other studies as well as studies performed by Mortazavi et al. which could not find any association between mobile phone use and the self-reported symptoms indicate the role of psychological factors in electromagnetic hypersensitivity [33,34]. Genotoxic effects of exposure to mobile phone radiation have also been studied. In a recent study, possible genotoxic effect of RF EMR (GSM, 1,800 MHz) in human lymphocytes was investigated through collaboration of six independent institutes. Genotoxicity end points were chromosome aberration, micronuclei, sister chromatid exchange and the alkaline comet assay. This study could not show any evidence of a genotoxic effect induced by RF EMR [35]. Regarding possible carcinogenic effects of mobile phone radiation, mobile phone users were not more likely to have been diagnosed with brain tumors compared with nonusers [36,37]. However, a nationwide cohort study in Denmark showed little evidence of an increased risk of skin cancer among the users of mobile phones [38].

### Beneficial effects of RF EMR

Dr. Sheldon Wolff In 1992 published his popular paper entitled “Is Radiation All Bad? The Search for Adaptation” [39]. He was globally famous for his studies on the stimulatory and beneficial effects of low dose ionizing radiation. At this time, considering intensely increase in using mobile phones (more than 4.5 billion subscribers around the globe), we should change Wolff’s question to a new query “Is mobile phone radiofrequency (RF) radiation all bad?” [40]. Nowadays, in some developing countries with poor infrastructure for landlines, mobile phone use has exponentially increased in the last decade. Interestingly, in some parts of the world, mobile phones are the main or even the only available telephone system.

Substantial evidence indicates that cells pre-exposed to low doses of DNA damaging agents such as ionizing radiation, ultraviolet (UV) rays, alkylating agents, oxidants and heat become immune to the detrimental effects of high doses of these agents or even similar agents. This phenomenon is usually referred to as “adaptive response”. Olivieri et al. in 1984 for the first time reported that pre-exposure of human lymphocytes to low doses of ionizing radiation induced an adaptive response as decreased susceptibility to chromatid break induced by a subsequent high dose radiation [41]. Although the mechanisms underlying the induction of adaptive response after pre-irradiation by a low dose radiation are not fully understood, it has been demonstrated that the improvement of DNA repair may be involved in this phenomenon [42,43]. Also it has been revealed that p53 might be a crucial mediator of DNA repair process after exposure to a low dose [44].

The induction of adaptive response phenomena by exposure to radiofrequency radiation as either increased resistance to a subsequent dose of ionizing radiation or resistance to a bacterial infection has been also reported [20,45-51]. The mechanisms of radiofrequency-induced adaptive response are not clearly known, so far [47]. It has been also recently shown that when laboratory animals are pre-exposed to electromagnetic radiofrequency radiation emitted from a common GSM mobile phone, they become resistant to a following bacterial infection [17,52]. Furthermore, there is another report by Plews et al. that indicated the induction of adaptive response induced by low-dose whole-body radiation treatments as prolonged survival of prion-infected mice by reducing oxidative stress [53]. As discussed in our previous articles, RF-induced resistance against bacterial infection can open new horizons in overcoming the problem of long term human stay in the space [54].

Furthermore, the possible advantageous effects of radiofrequency radiation are not only restricted to the induction of adaptive responses. Reaction time plays a critical role in performing activities necessary to better cope with life’s threats and/or avoid hazards. Reaction time widely varies from an individual to another, and increased reaction time may lead to fatal accidents. Previously, it has been indicated that the visual reaction time of university students was significantly decreased after a ten minute exposure to electromagnetic radiation in radiofrequency range emitted by a common mobile phone [19]. This finding is in line with the findings of other researchers who reported improved cognitive functions such decreased reaction time or improved performance on attention and short term memory after exposure to radiofrequency radiation [55-60]. Furthermore, it has been reported that the reaction time in radar workers whom are occupationally exposed to radar microwave radiations is significantly shorter than that of the control group [18]. Altogether, our results revealed that exposure to microwave radiation decreased the reaction time which helps people better respond to different threatening situations. Therefore these exposures can decrease the probability of human errors and reduce destructive accidents. Different trials [61-63] and some epidemiological studies [64,65] conducted over the past years were unable to reveal effects of exposure to mobile base stations on cognitive functions. Furthermore, stimulatory cognitive effects caused by long term exposure to RF radiation have been shown in some studies performed over the past years. Arns et al. in 2007 used a word interference test and reported that long term intense cell phone use caused better performance of normal individuals [66]. Furthermore, in 2009 Schuz et al. indicated that long-term mobile phone users (those who used 10 years or more) had a 30–40 percent reduced risk of hospitalization because of Alzheimer’s disease (AD) and vascular dementia [67]. Arendash et al. previously indicated that currently available drugs only treat/mask AD symptoms for about one year (none of these drugs directly slow or lessen AD pathogenesis). In this light, they proposed that high frequency electromagnetic radiation can be a safe, non-pharmaceutical approach to treat AD [68-70]. Recently, Arendash reported that as AD drugs cannot get into neurons and as most of these drugs have a single mechanism-of-action, pharmacologic interventions against AD seem to be unsuccessful [71]. Therefore, he stated that long-term transcranial electromagnetic treatment (TEMT) can prevent and reverse both cognitive impairment and brain amyloid-β (Aβ) deposition in AD transgenic mice. He also claimed that TEMT even improves cognitive performance in normal mice. Arendash believes that understanding the mechanisms of action of transcranial electromagnetic treatment (TEMT) can help scientists provide remarkable therapeutic methods for prevention and treatment of other neurologic disorders such as Parkinson’s disease, or injuries like traumatic brain injury and stroke [71].

## Conclusion

Substantial evidence indicate that pre-exposure to radiofrequency radiation can induce stimulatory phenomena such as adaptive response. Furthermore, it has recently been shown that pre-exposure of laboratory animals to RF EMR increases their resistance to a subsequent bacterial infection, a response which may have implications in manned deep space exploration. Interestingly, the potential beneficial effects of RF radiation are not only limited to the induction of adaptive phenomena. Our findings showed that human exposure to RF EMR leads to the better performance of short term memory and decreased reaction time. Other investigators have also provided evidence that confirms the induction of RF-induced cognitive benefits such as protection against cognitive impairment in Alzheimer’s disease. In this light, a challenging issue will arise whether there are other radiofrequency-induced stimulatory or beneficial effects. It is also challenging to investigate the possible applications of these RF-induced stimulatory effects. Further research is needed to clarify the health effects of short and long term effects of human exposure to different levels of radiofrequency radiation.

## Abbreviations

EMR: Electromagnetic radiation; RF: Radiofrequency; RF EMR: Radiofrequency electromagnetic radiation; SAR: Specific absorption rate; RMS: Root mean square; MHz: Megahertz; GHz: Gigahertz; GSM: Global system for mobile communication; UV: Ultraviolet; AD: Alzheimer’s disease; TEMT: Transcranial electromagnetic treatment.

## Competing interests

The authors declare that they have no competing interests.

## Authors’ contribution

All authors contributed significantly to the paper. The first three authors participated in data collection and prepared the initial draft of the manuscript. SMJM is the senior and corresponding author and suggested the research project. He also fully supervised the data collection. All authors read and approved the final manuscript.
